# Looping Disruption: A Relational Mechanism Enhancing Treatment Readiness among Individuals Convicted of Sexual Offending?

**DOI:** 10.1177/10790632231224380

**Published:** 2023-12-28

**Authors:** Stina Lindegren

**Affiliations:** 1 Department of Social Work, 151672 Uppsala University, Sweden

**Keywords:** sexual offender treatment, treatment readiness, looping disruption, total institution, motivation

## Abstract

Many convicted individuals do not enter or complete treatment programs in prisons, which limits effective rehabilitation and prevention of recidivism. Treatment readiness is suggested to be an important construct when addressing this problem. Nevertheless, the underlying processes (e.g., how readiness factors interact) are not well studied, and even less is known regarding readiness in the sub-population of individuals convicted of sexual offenses. This paper aims to open up the “black box” and explore psychosocial and context-specific processes behind treatment readiness from the vantage point of the individuals’ lived experiences. In-depth interviews were conducted with 19 adult men convicted of sexual offenses in Swedish prisons, treatment participants (N = 13) as well as non-participants (N = 6). The thematic analysis illustrates readiness obstacles in terms of unintended antagonistic forces in the correctional system operating in the opposite direction of rehabilitative objectives. Nonetheless, a hypothesized relational mechanism, *looping disruption*, initiated by a non-punitive and supportive response (from prison staff, therapists, close ones, or inmates) to the convicted individual’s negative behaviors or emotions, appeared to reverse such negative, punitive loops, contributing to the mobilization of treatment readiness. Implications for theory, policy, and practice are discussed.

## Introduction

Offender treatment participation is associated with decreased risk of re-offending (e.g., Landenberger & Lipsey, 2005; Lipsey et al., 2007), as well as improved reintegration into society (Mathlin et al., 2022). Moreover, for individuals convicted of sexual offending, there is support for treatment programs (e.g., Gannon et al., 2019; Hanson et al., 2009; Lindegren, 2023, forthcoming; Lösel & Schmucker, 2005) and its cost-benefits (Marshall & Marshall, 2021). Nonetheless, not everyone wishes to participate in treatment programs. The number of individuals convicted of sexual offending who accept to participate in treatment varies considerably, from 13% to 96% (Langevin, 2006; Marshall, 2021; Marshall et al., 2011). However, these numbers do not seem to differ substantially from interventions in other fields (Mann et al., 2013), such as medicine, where between one-third and one-half of patients tend not to engage in treatments (Melamed & Szor, 1999). In comparison with treatment completers, treatment dropouts and non-participants^1^ have six times higher risk of recidivism in violent and sexual offenses, compared to participants who complete the program (Seager et al., 2004). Accordingly, this group is of particular interest.

Internal motivation is often understood as crucial for treatment engagement (Prochaska & DiClemente, 1982), despite arguments that motivation is only one part of readiness (Bosma et al., 2017; Burrowes & Needs, 2009; Howells & Day, 2007; Ward et al., 2004). Hence, *readiness* is a wider, multifactorial concept referring to the preparedness of the client as well as the treatment provider, taking context and processes into consideration (Howells & Day, 2003; Ward et al., 2004). Howells and Day (2003, p. 320) provide the following definition of readiness: “Low readiness refers/…/to presence of characteristics (states or dispositions) within either the client or the therapeutic situation, which are likely to impede engagement in therapy and which, thereby, are likely to diminish therapeutic change”. Another issue with theories of internal motivation (e.g., Prochaska & DiClemente, 1982) is that empirical research on those who have sexually offended shows that low motivation at the time of treatment intake, surprisingly, does not predict higher levels of recidivism (Hanson & Morton-Bourgon, 2005). It is hypothesized that staff may not always assess internal motivation adequately. Such findings are possibly related to stigmatization and shame, which cause defense strategies where sincere remorse and motivation to not re-offend are not correctly assessed (Frost, 2016; Hanson & Morton-Bourgon, 2005; Marshall et al., 2009; Maruna & Mann, 2006; Ware & Mann, 2012). Individuals convicted of sexual offenses often display various cognitive distortions (denial, minimization, or excuses), which broadly can be placed into two categories. The first category comprises criminogenic, *offense-supportive cognitions,* which increase the probability of sexual offending, e.g. ideas of sexual entitlement, and thus should be targeted in treatment (Ward, 2000). The second category, *post-hoc cognitions* (e.g. denial), refers to coping strategies (against shame, e.g.) after the crime has been committed, argued to constitute normal, even healthy, reactions (e.g. Maruna & Mann, 2006; Yates, 2009). Accordingly, defensive strategies such as denial and other post-hoc cognitions, as well as how professionals respond to them, play an important role when trying to understand readiness among those who have sexually offended.

### Looping in Prisons as a Readiness Barrier

It may be the case that the institutions themselves, that is, legal and correctional systems, reinforce, or even produce, several of these post-hoc cognitions, or defense strategies, as argued by Ievins (2023). Hence, social dimensions are essential when trying to understand readiness, especially among individuals convicted of sexual offenses who are subjected to exceptional stigmatization (Tewksbury, 2012). Goffman (1961, p. 4) introduced the idea of *total institutions* as being relevant when studying closed places of residence cut off from the rest of society, such as prisons. *Looping* is a phenomenon in these institutions, described as “an agency that creates a defensive response on the part of the inmate takes [sic!] this very response as the target of its next attack” (Goffman, 1961, pp. 35–36). Thus, defensive, face-saving reactions (e.g., contempt, rudeness, or cognitive distortions) aroused by being incarcerated and the attacks upon the self that this experience may entail, often function as an expression of behaviors which the institution considers to need additional punitive measures. As I will propose in this paper, looping is a concept that can help improve our understanding of the psychosocial dimensions behind treatment readiness.

### Previous Research on Offender Treatment Readiness

Motivational interventions, such as motivational interviewing (McMurran, 2009; Miller & Rollnick, 2013), are central and popular features of offender treatment and preparatory programs (e.g. Marshall et al., 2008). Regarding the broader concept of readiness, the Multifactor Offender Readiness Model, MORM, is a multifactorial conceptual model explaining treatment readiness in the population of individuals convicted of offenses (Ward et al., 2004). According to the MORM, cognitive, affective, behavioral, external social factors (family and friends), context factors (location of treatment, etcetera), and volitional factors are all important to understand readiness. There is theoretical and empirical support for the model (Sturgess et al., 2016; Ward et al., 2004). However, research that seeks to empirically validate MORM is scarce, seems to mostly be quantitative, and the mixed method studies usually entail qualitative data that are poorly described (see Bosma et al., 2017; Sturgess et al., 2016, p. 1889). Moreover, studies that empirically examine the dynamic processes and interactions between the different factors in MORM, from a client’s perspective, are needed.

The extant literature indicates that treatment readiness can predict treatment acceptance (for an overview, see Bosma et al., 2017) and participation over time (Alward et al., 2020). Readiness is linked to subsequent treatment change, completion, and (reduced) recidivism (Sowden & Olver, 2017), although some studies have not been able to confirm a relationship to therapeutic change (Watson et al., 2018). It is noteworthy that, alongside readiness, treatment *engagement* is a frequent concept in the reviewed studies that lack consistent definitions. Empirical research often describes aspects of readiness as well as treatment completion or dropouts, which complicate reviews of the literature. Furthermore, empirical studies of readiness have mostly investigated community-based treatment programs within a North American context (Bosma et al., 2017), limiting the generalizability of findings.

#### Institutional Practices and Readiness

Research points to the importance of positively-oriented approaches. Although criminogenic risks and needs are central to rehabilitation (Bonta & Andrews, 2017), too much focus on risk factors may not engage individuals who have offended sufficiently (Bosma et al., 2017; Youssef, 2022). Instead, supportive staff attitudes and de-labeling features have been suggested as central in the readiness process (using prosocial labels, rather than “criminal” or “sex offenders”) as well as the focus on personal future goals (Stasch et al., 2018; Sturgess et al., 2016; Willis, 2018). Studies that explore reasons stated by individuals convicted of non-sexual offenses to engage in treatment are scarce, even fewer specifically on the population of those convicted of sexual offenses (Sturgess et al., 2016). The findings from a systematic review of 13 studies (qualitative, quantitative, and mixed methods) on reasons stated by individuals who have offended for treatment completion or non-completion indicated barriers to treatment acceptance, including denial, poor self-efficacy, negative views of treatment and expected outcomes, lack of choice or control, lack of perceived support from staff, and not feeling safe. Only one study in this review (Sturgess et al., 2016) included individuals convicted of sexual offenses (Mann et al., 2013). Low motivation, nevertheless, may also partly be understood as a feature of some individuals’ personality patterns or disorders (Alemohammad et al., 2017; Howells & Day, 2007).

#### Readiness among Individuals Convicted of Sexual Offending

##### External Motivation

Offender rehabilitation, in general, usually entails some level of coercion or strong incentives to participate in treatment. Research on how the level of coercion or voluntariness impacts treatment acceptance among those who have sexually offended are mixed, and confounders can obscure the picture. Court mandated treatment seems less favorable to voluntary entry (e.g., Levenson et al., 2023; Parhar et al., 2008; Seto et al., 2022). Nevertheless, for some individuals who have sexually offended, positive pressure from family members or the court might contribute to a wish to prove a willingness to change and paying a debt to society (Brown & Tully, 2014; Collins et al., 2010; Drapeau et al., 2004). While active encouragement from therapists can also serve as positive pressure to engage in treatment, negative pressure (perceived coercion), however, seems to reduce readiness (Bacharz, 2008; Kolton, 2002; Mann et al., 2013; see also Sturgess et al., 2016). Studies suggest higher motivation, different psychiatric conditions as well as external factors, such as opportunities for parole and inmate privilege, correlate with treatment participation among those who have sexually offended (Clegg et al., 2011; Jones et al., 2006; Langevin, 2006; see also Drapeau et al., 2004; Sturgess et al., 2016).

##### Internal Motivation

Internal motivation is strong among some individuals convicted of sexual offenses, comprised by a will to not re-offend and address issues perceived as related to their crime, such as depression, isolation, and own sexual abuse victimization background (Collins et al., 2010; Cooper & Holgersen, 2016). However, such client perspectives on readiness are often “bi-products”, only briefly discussed in studies focused on treatment experience. Regarding non-participants (or “refusers”) among those who have sexually offended, competing priorities (jobs or relationships) (Mann, 2009) are highlighted, as well as not perceiving one’s offending behavior to be wrong. Mann et al. (2013, p. 191) state that non-participants seemed to be “less aware of the effectiveness of treatment, reported seeing negative side effects of treatment in others and felt they had a higher social status in prison which could be damaged by attending treatment” (see also Brown & Tully, 2014). Denial of crime is sometimes associated with lower motivation for treatment (Jung & Nunes, 2012) and lesser likelihood of treatment participation (Langevin, 2006). Among individuals convicted of sexual offending who previously denied their offense, 67% reported fear of losing the support from family and friends as the main reason for denial (Lord & Willmot, 2004). Nevertheless, Mann (2009) argues that fear of social losses seems to be related to denial rather than to non-participation. In summary, few studies have examined the individuals’ own accounts regarding reasons for treatment participation or non-participation (e.g., Mann et al., 2013); furthermore, adequately described qualitative, process-oriented studies exploring psychosocial and context-specific dimensions and mechanisms behind readiness are needed.

## Conceptual Framework: Looping

The analytic tool employed when interpreting the empirical material in this study is the concept of *looping*. According to Goffman (1961), total institutions, e.g. prisons, have certain common characteristics. They give rise to a process of mortification of the self as a result of de-humanizing and degrading practices, such as being stripped of one’s personal belongings or assigned an inmate number, instead of the personal name. Goffman terms the defensive, face-saving reactions to this mortification process, and the controlling, punitive responses from the institution, looping (not to be confused with Hacking’s [1996] concept looping effects). As previously mentioned, looping is described as “an agency that creates a defensive response on the part of the inmate takes [sic!] this very response as the target of its next attack” (Goffman, 1961, pp. 35–36). Looping is related to stigma (Goffman, 1963), labeling theory (Becker, 1963), and concepts like gaslighting (Stern, 2007). Nonetheless, the punitive and controlling responses to the negative behaviors within total institutions is a defining feature of looping. Looping does not imply that the prison staff deliberately are acting out in punitive responses. Rather, they might be under the influence of moral and political pressure (Ware & Mann, 2012; Youssef, 2022). Thus, their interpretation of the inmate’s behavior as “deviant” is socially constructed and they presumably believe that punishing the inmate is their responsibility and that it in fact is in the inmate’s best interest.

Contemporary research on looping in mental health settings concentrate on how actions among patients are interpreted as signs of their mental illness and how official diagnoses as well as informal organizational labels are used to control patients (Alexander, 2006; Dobransky, 2011). It is conceivable that similar looping processes, such as inmates’ externalizing behaviors being interpreted as evidence of their “aggression problems” or “antisocial personality patterns”, occur in modern correctional services, which may obscure contextual factors. It is noteworthy that even problematizing lack of motivation or readiness, as in this paper, may constitute some form of looping, given the variability in recidivism risk, not all individuals who have sexually offended are in need of risk reducing treatment programs.

I claim that looping is highly relevant when studying individuals convicted of sexual offending, and that in fact, looping occurs not only in total institutions (prisons), but in society, as well as in, at least some of, the offense-specific treatment programs. These looping processes create an ongoing, vicious circle – a barrier to rehabilitation. Individuals who have sexually offended are often punished for “normal” (logical and understandable) behaviors for someone classified as a “sex offender”. On a societal level, stigma and fear constitute obstacles to help-seeking and preventative actions (defensive response) (Levenson et al., 2017). The public often view these individuals as predatory monsters, recidivists (despite evidence), unable to change, and public harassments and violent attacks against them are evident (Bonnar-Kidd, 2010; Harper et al., 2017; Tewksbury, 2012). Thus, this societal response (attack) creates further fear and internalized stigma, exacerbating the looping processes.

Regarding looping in legal systems and prisons, Ievins (2023) argues that the prison sends a stigmatizing message (c.f. Braithwaite, 2020) of individuals who have sexually offended as bad, manipulative “sex offenders” (the prison’s attack), which Ievins (2023) claims actually produces some of these individuals’ cognitive distortions (defensive response). An additional defensive response to this stigmatizing message is that they do not engage in any actual constructive moral work; rather, they focus on resisting the sex offender label (Ievins, 2023). Furthermore, some cognitive distortions can be interpreted by prison staff as being “in denial”, that is, lacking insight, which, despite the absent evidence of such an approach (Hanson & Morton-Bourgon, 2005; Ware & Mann, 2012; Yates, 2009), is further punished and targeted in some treatment programs (Ievins, 2023; Levenson et al., 2023). The internal hierarchies among inmates in prisons also produce looping. The fear of violence and harassment and mistrust this creates among individuals who have sexually offended, who are lowest in the hierarchy (Ugelvik, 2015), which can lead to a defense strategy of treatment refusal, may render attacks from the institution in the form of withdrawal of inmate privileges. Accordingly, looping can create a negative spiral, supposedly interfering with readiness and rehabilitative objectives (cf. Youssef, 2022). Looping relates to the external conditions outlined in the MORM, such as the negative impact of anti-therapeutic environments. However, as far as I know, the specific processes of looping have not yet been elaborated on in the MORM or offender readiness research in general.

### The Present Study

Although empirical studies support the idea of looping in prisons for those who have sexually offended (e.g., Ievins, 2023; Kruse, 2020), readiness studies seldom engage with this concept, which may constitute a readiness barrier. In addition, qualitative exploration of underlying psychosocial processes and mechanisms behind readiness is lacking. Such research can improve our understanding of readiness. Furthermore, few studies have examined the views of those who have sexually offended regarding the issue of participating in treatment or not. This study seeks to fill these gaps by a qualitative in-depth examination of the accounts of individuals who have sexually offended, using interviews and an interpretative, meaning-making approach guided by Goffman’s concept of looping. Accordingly, the aim of this paper is to unpack the psychosocial and context-specific processes and mechanisms behind treatment readiness among these individuals, from their lived experience. The research question guiding this study was: How do incarcerated individuals convicted of sexual offending experience potential obstacles and facilitators for participation in treatment programs?

## Methods

Since the aim of the study is to explore readiness from a perspective of lived experience, this study employs a qualitative in-depth interview design. Data analysis and theme construction relied predominantly on a latent analytical focus; hence, underlying readiness processes were interpreted to a high extent by analyzing implicit meanings in the data. The study is part of a larger research project examining various aspects of treatment as well as previous help-seeking among Swedish men who have sexually offended, drawing primarily on comprehensive interview data (see Lindegren, 2023, forthcoming). Ethical approval was obtained from the Swedish Prison and Probation Service as well as the Swedish Ethical Review Authority (2020–04038) in accordance with the Swedish Ethical Review Act (SFS, 2003:460).

### Recruitment of Subjects and Sampling

Purposive sampling was employed; thus, those who had completed a sex offender treatment program as well as those who had not participated (and were negative or hesitant to treatment) were eligible for inclusion. Individuals under the age of 18, those at high risk of displaying violence towards professionals, as well as those with serious reality-altering mental conditions, were excluded. The concept of information power (Malterud et al., 2016) was used to plan for an adequate sample size. Prison staff recruited interviewees. Interviewees did not receive financial compensation, and participation was voluntary. Written informed consent was obtained, and information stating that participating (or not) in the interviews would not affect their sentence conditions was provided in written form as well as orally.

### Sample

The sample comprised 19 incarcerated adult men convicted of sexual offending in prisons representing all security levels (low, medium, and high). Background characteristics were collected from a self-reported questionnaire. Age ranged from 26 to 82, and the most common offenses, 84% of the cases, were rape of adult or child, including aggravated. Six participants had at least one non-sexual offense under the same sentence, most commonly assault. Six interviewees had multiple sexual offenses under the same sentence, four had a previous sexual offense sentence, and eight of them had any previous criminal sentence. Sentences ranged from six months to nine years (Md = 5 years). There were equal numbers of child versus adult victims; moreover, most of them were closely related to the study participant. Only two interviewees, treatment-participants, reported sexual interest in children. Most interviewees had children, and half of them a steady partner relationship. Five of them were on sick-leave or unemployed before imprisonment, and the rest worked or studied. Fourteen had a secondary school degree or higher education. All interviewees spoke fluent Swedish, except two who had quite recently immigrated to Sweden.

The group of treatment-participants (N = 13) were more homogenous than the non-participants (N = 6), in terms of them all admitting guilt of their offense, and no one had low recidivism risk (cf. half of the non-participants were low risk assessed and, thus, probably not always prioritized for treatment). Everyone reported being very pleased with the treatment program; however, several described having had hesitations prior to treatment. Among the non-participants, motivation/approach to treatment varied, from being strongly negative, hesitant, to ambivalent. One was very positive to treatment but was not offered participation.

### Rehabilitative Context

Sweden is a rich, highly gender equal, western country with strong egalitarian values. The state-governed Prison and Probation Service, in international comparison, has humane prison conditions and is more oriented toward rehabilitation than punishment, a phenomenon referred to as *Scandinavian exceptionalism* (Pratt, 2007). However, some researchers argue that there is a current punitive turn in Scandinavian criminal policy (Storgaard, 2022). Sexual offense legislation is comprehensive, and the definition of rape is wider than in most countries, including all non-consensual acts comparable to sexual intercourse (Holmberg & Lewenhagen, 2020). Less than a third of all individuals convicted of offending participate in treatment programs (Kriminalvården, 2022). Participation in sex offender programs is free of charge and is not mandatory for incarcerated individuals; however, there are strong incentives to participate in order to receive different privileges. The program offered to the interviewees is manual-based, but flexible, and based on cognitive behavioral therapy and the risk-, need-, and responsivity model (Bonta & Andrews, 2017). The aim is to reduce the risk of recidivism. Those who deny guilt of crime are included in treatment, provided acknowledgement of any risk relevant problems the program targets (denial is not a treatment target). Program-therapists are predominantly psychologists or social workers employed by the Prison and Probation Service (Lindegren, 2022).

### Interviews

Interviews were conducted in four prisons (a few via telephone due to COVID-19) from June 2021 to July 2022. A semi-structured guide was used for the interviews, which were guided by an experiential/phenomenological approach (Aspers, 2009; Braun & Clarke, 2022). The interview guide included topics concerning personal background, the decision process regarding participation (or not) in treatment program, and thoughts on what matters in general when offering sex offender treatment, including barriers/facilitators to treatment and what role family and society may play in this process. The non-participants were also asked questions regarding their views on potential needs or support other than sex offender treatment. Questions were primarily open, for example “Can you describe the first time you received information about the opportunity to participate in the treatment program and what you thought and felt about this?” and “Did you talk to friends/family about the decision, if so, how did this affect your decision?”

The interview procedure was inspired by *teller-focused interviews*, suitable for sensitive topics (Hydén, 2014), emphasizing relationship aspects and the interviewee’s own narrative. The interviewer has years of experience in probation work and offender treatment, predominantly with individuals convicted of sexual offending. This insider-perspective (Hayfield & Huxley, 2015) was perceived to have facilitated the production of rich data as well as interviews characterized by trust. Nonetheless, there is an inherent asymmetrical power situation when researching incarcerated individuals. The outsider position of being a non-convicted, white, Swedish-born woman may have entailed challenges to fully comprehend the effects of marginalized identities among the interviewees.

### Data Analysis

The interviews were on average 90 minutes long, audio recorded, and transcribed verbatim. Notes were taken for initial reflections. Data were inductively coded and analyzed in NVivo, assuming a critical realist perspective. Data relevant to the research question were coded and relied on both latent (implicit meaning) and semantic (explicit meaning) information. There was a hermeneutic analytical procedure where all interviews were coded separately and subsequently analyzed across the data set. Hence, analysis oscillated between details (individual experience and meaning) and the whole (shared meaning). In total 55 codes were subsequently reduced to two themes containing two sub-themes each. Some codes related to internal motivation are briefly summarized in the analysis, since the final analysis does not focus on non-relational internal motivators.

The data analysis was initially exploratory, with the starting point of the research questions, and guided by principles of reflexive thematic analysis (Braun & Clarke, 2022). Hence, data relevant to the research question were coded and patterns of shared meaning, regarding readiness, across the data set subsequently formed the two themes, and sub-themes. As the themes were constructed and theory was incorporated into the analysis, it became apparent that the contribution of the study was primarily of conceptual nature. The analytic procedure was somewhat similar to a grounded theory approach. Theory was applied after having finalized coding and initial theme development, aiming to explain the patterns generated from the data (abduction). Accordingly, the content in the initial themes, describing institutional readiness barriers and facilitators, seemed to be theoretically explained by Goffman’s concept of looping. This gave rise to the conceptual development of “looping disruption”, which was used to interpret and name the second theme, describing the relational readiness facilitative process.

The author conducted and analyzed all interviews. Using a single coder may risk the occurrence of bias, accordingly, actions were taken to ensure rigor and trustworthiness. The author used a reflexive journal and took notes throughout the entire research process. Furthermore, content of the reflexive journal as well as coding, themes and theoretical analysis were discussed in multiple collegial fora. Interviewees are de-identified and some details have been modified to prevent identification. Some wordings in the quotes are slightly adjusted to enhance readability.

## Results

The aim was to study processes; thus, excerpts from a few interviewees are more frequent since this arguably illustrates processes better than fragmented excerpts from many individual processes. The two main themes, including two sub-themes each, represent readiness obstacles and readiness facilitative processes; see table 1. Names are fictitious.

**Table 1.** Themes and Sub-Themes.

Looping barriers	Expectations of better sentence conditions
	Contagious fear and mistrust in prison
Looping disruption	Participating for, but not with, close ones
	The crisis – a unique, relational opportunity

### Looping Barriers

This theme illustrates the readiness obstacles primarily related to the criminal justice system and antagonistic forces operating in a non-rehabilitative direction, illustrating looping processes as well as study participants’ strategies to manage, or avoid, looping (Goffman, 1961).

#### Expectations of Better Sentence Conditions

Several interviewees stated that they, at least initially, participated in treatment, or contemplated participating, in order to be compliant with the Prison and Probation Service. Thus, following all the rules and expectations was an adaptive strategy to avoid controlling and punitive responses (i.e., looping), which Goffman calls “playing it cool” (1961, p. 64). In addition, external motivators related to their sentence conditions, e.g., being able to have visitors or conditional release earlier, were important.

Before investigating the role of the external motivators for readiness, it is noteworthy that the interviewees often highlighted an individual’s internal motivation as most important, especially in the accounts of the treatment-participants. The most common explicitly stated reasons for entering treatment was a will to not re-offend or hurt others, to receive insights, regain control over their life and behaviors, as well as a desire to take responsibility (cf. Maruna & Mann, 2006). When having completed treatment, these were perceived as the most significant outcomes of treatment (Lindegren, forthcoming, 2023). The most frequently stated reasons not to enter treatment were being convinced that one would never re-offend, incompatible goals (being more concerned about finding a job, housing, economy, etcetera), not wanting to discuss the offense with others, or perceiving to not have done anything wrong. These may be valid reasons for declining treatment; however, some of the interviewees’ accounts were contradictory and included information about events which could be understood as, at least partly, contributing to treatment hesitation. These latent processes are the focus of this paper.

Several interviewees claimed that the “rewards” they expected regarding their sentence conditions post treatment failed to appear. It is not known whether their expectations were legitimate, nonetheless, the interviewees expressed confusion and disappointment about the absence of rewards. Treatment-participant Josef, a young man convicted twice of sexual offenses, said he was so disappointed that he declined participation in the research interview. However, later, he changed his mind, seemingly because he realized he was not deceived by the Prison and Probation Service as he had initially thought (he described a conversation with his therapist that seemed to clear some misunderstandings; thus, perceived looping was disrupted):

Josef: I started the program, and I had a few visits, with no remarks. Then I thought, ‘What’s happening?’ I thought about what was said before, ‘you will have visits’, but after the program [this did not happen]. Did I do something wrong? Do you see what I mean? Did I SAY^2^ something wrong in the program? I was devastated. […] They told me prior to this that I WILL have unsupervised visits. But after the program, it was the opposite! And then I thought, did [therapist’s name] say something about my participation?

The sub-theme illustrates the importance of external readiness factors delineated in MORM as a means to avoid looping, such as withheld privileges, where a perceived lack of institutional rewards in this case seemed to produce mistrust and a risk of undermining the motivational power of external incentives.

#### Contagious Fear and Mistrust in Prison

Data revealed experiences of mistrust and fear. These are normal defensive reactions to the mortification process in a total institution (Goffman, 1961), which, however, may be subjected to punitive responses and thus constitute another example of looping, as well as a consequence of looping. Fear and mistrust seemed to flow, and sometimes exacerbate, in several systems within the correctional context between (some) inmates, in (some) staff-inmate-relationships, with regard to (some) experiences with routines in prison, such as risk assessments, etcetera. In fact, several interviewees discussed other inmates’ mistrust toward me as a researcher as well. There was a fear of looping, where talking about their offense was thought to result in controlling measures, such as data ending up in the hands of the police.

The internal inmate hierarchies were obvious (cf. Ugelvik, 2015), creating a readiness barrier, especially for some of the study participants. Young treatment-participant Robin had initially been hesitant to participate in treatment due to the group format.

Robin: If there is someone in the program convicted of ‘standard rape’ and then I show up, convicted of a pedophilia offense, then he might have very strong opinions about what I have been convicted of. So, there were actually risks in doing so.

When asked about the risks, Robin continues: “Well, that I would be beaten up. Or that information would spread, that could lead to one being ostracized.” In addition to fear of negative side-effects, there seemed to be frequent misperceptions about the treatment:

Hasse: There were many at [a previous prison] who said: ‘No, I don’t want to participate in SEIF; that’s a rape program.’ I [Hasse] said: ‘It’s a program, but it’s not JUST about rape; it’s about a lot of other things that are useful and good.’

For those who completed treatment, they stated, in accordance with Hasse, that their fear of negative side effects was not confirmed (Lindegren, 2023 forthcoming). According to the interviewees, many inmates seemed to believe that participation in treatment would include a mandatory acceptance of every statement, fact, or circumstance described in the official verdict and records as 100% true (a controlling response, i.e., looping), which is not the case for this specific treatment program. This is an indication of not only the looping avoidance strategies but also of the intervention stigma (cf. Madden, 2019).

Some interviewees’ accounts indicated other paradoxical, antagonistic features of the correctional system in general, which seemed to punish vulnerability and reward violent behavior, creating or exacerbating mistrust. Data demonstrated substantial general mistrust or even bitterness toward the criminal justice system. Mistrust can be related to personality characteristics or childhood adversity experiences, common in the population of those who have sexually offended (Jespersen et al., 2009; Stinson et al., 2023). In the current study, the accounts seemed related to experiences of lack of procedural justice; arbitrary and unjust responses where the interviewees felt further accused and attacked during the trial as well as within the correctional system (cf. Ievins, 2023). They felt that some prison staff were not supportive, possibly due to the interviewees’ defensive, face-saving stance, interpreted as being “non-cooperative” or “in denial”. Additionally, a few of the interviewees claimed that expressing mental health issues or depressive thoughts did not lead to support; rather, they could strangely find themselves placed in solitary confinement. These phenomena, where normal reactions (e.g., depression) and self-defensive strategies are punished, can be understood as expressions of looping (Goffman, 1961). Treatment-participant Eric, who had a long history of violent behavior, now convicted of raping his girlfriend, described an unusual strategy he adopted in order to be placed in a, what he perceived as, rehabilitative-oriented (high security) prison.

Eric: I had two options: either coming here [rehabilitative-oriented prison] or to [other high security prison] and I knew that if I go to [other prison] then I will get worse. Because I heard you will not receive help there. So, I started picking fights with people [inmates] who were going there [other prison].

Stina: Ok, so you consciously got into fights…?

Eric: Yeah, exactly.

Stina: … so that you would be placed somewhere else?

Eric: Yes, I told them that [slightly embarrassed laugh], a friend of my family worked at [other prison] and that I wanted to go there. So, then, I was able to come to [rehabilitative-oriented prison]. I know how the Prison and Probation Service works. If I had said I wanted to come here, then they wouldn’t have let me come here.

Hence, due to mistrust, that the explicit request for a certain prison would not be met, Eric seemingly “used” looping to his advantage. From experience, he knew that frustration and face-saving strategies, expressed as externalizing, violent behavior in prison, render controlling and punitive measures, such as being placed in a high security prison. For security reasons, parties involved in a fight are sent to separate prisons. Furthermore, being placed in a prison where a friend works constitutes a risk for escape, etcetera, which results in controlling measures.

Similarly, non-participant Eskil described a meeting with a psychologist. He had engaged in a several weeks long, systematic assessment initially in his sentence, where he wanted to discuss some questions (according to his accounts during the interview, these questions contained several victim-blaming cognitive distortions regarding his offense):

Eskil: And the only reply I received from her was ‘you are good at lying’. I never found out in what WAY I lied. But that was right at the end that we talked about it; if I had done it earlier, I would probably have just said ‘let’s skip this’.

This also constitutes an example of looping, where a defensive reaction (cognitive distortion) evokes a punitive response (called a liar). Eskil’s story indicates that his question seemed to have triggered the professional’s confrontational response and reduced readiness. The theme demonstrates the importance of external readiness conditions: the emotional dynamic in prison (fear and reciprocal mistrust), looping and looping avoidance strategies, thus, processes that seemingly operated in a direction opposite of what the Prison and Probation Service probably intended.

### Looping Disruption

**Figure 1.**
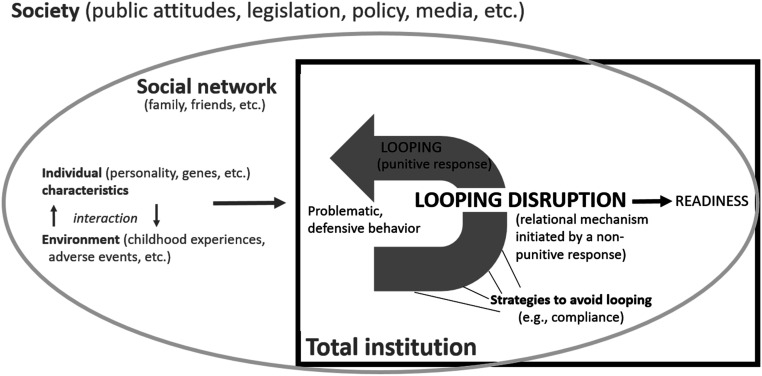
Looping disruption. *Note.* Looping disruption situated in the context of readiness factors.

The second theme underscores the relational processes that seemed to facilitate treatment readiness, conceptually understood as what I term *looping disruption*: a mechanism initiated by a non-punitive, supportive, authentic, and caring response (from staff, therapists, other inmates, or family) to the convicted individual’s negative behavior or emotions within the total institutions, which reversed looping and enhanced readiness; see Figure 1.

#### Participating for, but not with, Close Ones

One fundamental motivator was the interviewees’ families, including parents, children, partners, and other relatives. They described that they participated *for*, but not *with*, their close ones; thus, treatment participation was seldom explicitly discussed with their close ones. Also for non-participants, family often appeared as the fundamental value in their lives, emphasizing personal identity readiness factors (Ward et al., 2004). Robin, who had molested a closely related child, and, as described previously, hesitated because of the group format, says:

Robin: What made me want to do it anyway [despite hesitation] was that, partly then, the expectation that I should do this and partly because I knew it would be good for me, regardless. I thought to myself, would I be able to say, when I get out, to my parents, for example, ‘no, I didn’t participate because it was group therapy?’ They wouldn’t have handled that well at all. […] I felt like I kind of owed people to do it. I have to work on this.

Thus, bonds with close ones can constitute an implicit, mental form of positive pressure, supporting the person to overcome fear and mistrust and disrupt looping, thus preventing punishment (suspended privileges) if he does not engage in treatment. Nevertheless, this resource did not seem to be optimized; hence, explicit family involvement in the treatment decision process could possibly enhance readiness further.

#### The Crisis – a Unique, Relational Opportunity

Treatment acceptance was often described as stemming from the life crisis, initiated by being convicted of a sexual offense and being incarcerated, and the way that crucial social interactions and trustful relationships mobilized treatment readiness, despite ambivalence or hesitation, through looping disruption. Eric describes this window of opportunity, opening up in a conversation with a correctional professional, subsequently followed by the previously mentioned violent strategy to receive rehabilitation. Eric was placed at an assessment unit where a comprehensive psychosocial assessment was performed in order to determine his risks and needs, as well as appropriate prison placement:

Eric: That was my turning point, why I really decided. It was when I met my assessor at the end [of the assessment placement]. Because it was just chaos there. I was in fights, going in and out of solitary confinement, and I didn’t care about anything. But then, I met my assessor. He was good, and at the end, on the last day, I will never forget this, he shuts the file, looks at me and says he has seen similar cases like mine, people with violence, die, they die [inaudible] from violence and so on. And then he says: ‘They come here and do their sentence and a few years pass, then they get out and then they come back and they sit here for life. So, I really hope you deal with this now.’ And I kind of lay there, thinking about this for a very long time. And I thought: now you are at a crossroads; either you take that road, or the other. And that’s where I decided to just deal with it all. […] I have already been extremely lucky that I haven’t killed anyone. Honestly, I’ve had friends die on the spot. When he said that, it was an eye opener. I saw that, in the future, it would not only affect me; it would affect my children, my family, that person’s family. And I don’t want that on my conscience. So that’s why I sort of chose to deal with it. That was the only thing. Because he was good, he was cool, and I noticed he said so because he cared. He wasn’t just doing his job.

Similarly, non-participant^3^ Ardalan, who, like Eric, had a long non-sexual criminal record, described his initial strong resistance to treatment, including a rude attitude toward the therapist trying to convince him to participate. Ardalan had participated in the precursor to the current treatment program during a previous sentence, and felt degraded by the therapist, whom he described as very confrontational. He claimed that normally, the word psychologist gives him “the shivers”:

Ardalan: But then I thought, ‘hey, he is sitting here and listening when I’m pouring out my guts to him. He still gets his paycheck; he could just get up and leave.’ But it was something with [therapist’s name] that made me begin to like him. I think he is very sharp at what he is doing.

Eric’s and Ardalan’s stories appear as illustrations of looping disruption. The professionals did not seem to interpret the externalizing behaviors as antisocial expressions in need of punitive correction. Instead, they recognized Ardalan and Eric as subjects with agency and insisted on promoting rehabilitative measures, where elements of trust, concern, care, honesty, and authenticity, namely therapist’s characteristics consistently demonstrated as important for therapeutic change, were apparent (Marshall, 2005; Wampold, 2015).

Supportive relationships among inmates also seemed to promote looping disruption, since fear, that is, not daring to participate in treatment, may render punitive responses (withheld privileges). As previously described, Robin was uncomfortable with the group format; however, a meeting with a fellow inmate changed this:

Robin: He was just very easy to talk to, very trustworthy. Very good at listening and so on, so I had confidence in him straight away. So we became friends and we sat solving crosswords in his room and just talking about everything and then, finally, he asked me what I was in for. I said I didn’t want to tell, but he said it was ok. Then, I felt I wanted to do it. So I told him and then we sat up until 4 o’clock in the morning or so, and talked. So that was quite a long time. He recommended that I participate. We talked several times about it until I finally said yes. And that was a very good decision. I ended up in a very good group.

This theme illustrates how personal identity readiness factors, where close ones appeared as a fundamental value, and timing readiness factors appear to be closely intertwined with social support readiness factors (Ward et al., 2004), intersecting at the proposed relational mechanism: looping disruption. Looping barriers were experienced by interviewees from both groups, whereas looping disruption, as one would assume, was a phenomena prevalent in the accounts of the treatment-participants.

## Discussion

The psychosocial and context-specific processes and mechanisms behind treatment readiness among those who have sexually offended are largely unexplored. The present study illuminates, in particular, the relational and context-bound processes that produce obstacles to, and those that facilitate, treatment readiness by employing and developing Goffman’s (1961) concept of looping in total institutions. The analysis demonstrates readiness barriers where antagonistic forces (looping) in the correctional system operated in the opposite direction of rehabilitative ideals. Nonetheless, a hypothesized relational mechanism, *looping disruption*, initiated by a non-punitive and supportive response to the convicted individual’s negative behaviors or emotions, appeared to reverse such negative, punitive loops, and enhance treatment readiness.

### Implications for Theory

I introduce the term *looping disruption* to describe the hypothesized relational mechanism illustrated in this study. Looping disruption is a proposed mechanism initiated by a non-punitive, supportive, authentic, and caring response (from prison staff, therapists, close ones, or inmates) to the convicted individual’s negative behavior or emotions, which reverse looping. Looping disruption stresses that readiness can be mobilized in the everyday interaction, not just in preparatory programs or motivational interventions. It is not necessarily a phenomenon restricted to the actions of therapists or other professionals. It is about significant human encounters; hence, all individuals can play a role in the readiness process. Being seen and treated as a subject, not as an “inmate” or a “sex offender”, appears to break the cycle of mistrust and fear and facilitate agency. The concept of looping disruption also highlights that these constructive encounters are important, particularly in total institutions, since they, paradoxically and unintentionally, might undermine readiness processes.

Looping disruption can serve as a means to understand one, of probably several, readiness mechanisms, placed in the category of *individual action mechanisms*, hence, psychosocial mechanisms operating on a micro-level (Hedström & Swedberg, 1996, p. 297). Associated concepts can describe certain parts of the process, such as the therapeutic alliance (Wampold, 2015) and Goffman’s original concept of looping. However, none of these concepts provide a fine-grained explanation which includes the whole chain of events and interactions between contextual, psychosocial, and relational factors in the process in which the proposed mechanism lies and should be understood (Hedström & Swedberg, 1996). Looping disruption could be viewed as a (micro-) turning point (cf. Carlsson, 2012; Lindegren, 2023 forthcoming), thus, as an ordinary event in prison life, which may play a crucial role in readiness and initial desistance. However, turning points is a criminological concept (applied in various ways) with quite a wide scope, which does not fully encompass the specifics of the narrower relational mechanism proposed in this paper (Hedström & Swedberg, 1996). Goffman did not discuss how looping may be reversed; hence, further conceptual developments are warranted. Despite the theoretical and empirical support for comprehensive multifactorial models, such as the MORM (Sturgess et al., 2016; Ward et al., 2004; see also Burrowes & Needs, 2009), they often do not fully open up the “black box,” that is, provide a detailed explanation of the specific mechanisms or interactions between the different factors (Hedström & Swedberg, 1996; Maruna & LeBel, 2010). Accordingly, looping disruption might bridge some conceptual gaps and contribute to a hypothesis about how different factors in MORM interact.

### Implications for Policy and Practice

Needless to say, I do not suggest that violent or inappropriate behaviors among incarcerated individuals should never render sanctions or measures other than rehabilitative ones. Utilizing looping disruption to mobilize readiness just proposes wider interpretations of behaviors that consider the contextual factors present within a total institution, as well as societal factors, such as stigma. Negative behaviors may sometimes warrant corrective as well as supportive, rehabilitative responses. The concept of looping disruption can provide practitioners with a “readiness tool” to disentangle treatment-avoidant behaviors, both prior to treatment and during the treatment process. It can support the identification of defensive, face-saving reactions related to the mortification process (Goffman, 1961), prevent a punitive approach limiting readiness, and signal when and how supportive, motivational approaches should be intensified.

Although looping and the effect of contextual factors on treatment readiness among those who have offended in general, probably, are, at least partly, similar across sub-populations,^4^ this study indicates that there may be specific challenges to effective responses to readiness among those who have sexually offended, due to the internal hierarchies in prison and exceptional stigma surrounding sexual offenses (Tewksbury, 2012). Cognitive distortions are often challenging and provocative for professionals. Some cognitive distortions could, however, be interpreted as normal reactions when being convicted of a sexual offense (Ievins, 2023; Maruna & Mann, 2006). It can even be seen as an invitation to professionals (Todd-Kvam et al., 2019). Nevertheless, despite this, professionals sometimes punish individuals with these reactions (looping). This places the individual in a catch-22 situation (cf. van Oorschot et al., 2017), where they withdraw from interaction with staff (Kruse, 2020). A possible punitive turn in criminal policy (Storgaard, 2022) may propose that individuals who have offended (not the least those who have sexually offended) should indeed experience a strong punishment. Despite the lack of evidence for such approaches, there still seems to be negative attitudes (Harper et al., 2017) supporting the importance of overcoming denial or excuses, possibly due to a sense of political and moral pressure to not be “soft” on those who have sexually offended (Grady et al., 2022; Levenson et al., 2023; Youssef, 2022). Nonetheless, this study corroborates research suggesting that additional punitive, stigmatizing experiences, beyond being convicted and sentenced, such as being labeled a “bad person” or a “liar” in correctional facilities (looping) (like in Eskil’s case) may actually reduce readiness and thus be counterproductive (e.g. Braithwaite, 2020; Ievins, 2023; Maruna & Mann, 2006; Tewksbury, 2012). Accordingly, since looping disruption implies that promoting readiness is not solely the task of therapists, but all staff who encounter individuals convicted of sexual offending, correctional services should invest in specialized training for prison staff too. Correctional staff should receive training in the stigmatizing social context of sexual offending and cognitive distortions as a defense mechanism or legitimate treatment need. Thus, such behaviors should not render punitive or dismissive reactions (looping), but instead intensified supporting responses in order to utilize these cognitive distortions to mobilize readiness and, thus, increase treatment participation.

Regarding external incentives, several of the treatment-participants stated that their motivation shifted from mainly external to internal during the treatment process (Lindegren, 2023 forthcoming), which suggests external motivators are not necessarily sub-optimal. Nonetheless, as indicated by the present study, attention should be paid to how external incentives are employed. Not receiving expected “rewards” can undermine trust and reduce the cooperative alliance with professionals. Standardized risk assessments sometimes conflict with other institutional messages that signal participation in treatment programs will automatically lead to better sentence conditions (cf. Ugelvik, 2022). When they do not, mistrust can spread even further in the correctional system. Sincere and clear communication about the possibility of rewards, therefore, should be carefully considered to avoid misperceptions.

The findings in this study are consistent with the central aspects of the MORM. It also corroborates empirical findings, such as the occurrence of competing priorities (Mann, 2009), the importance of internal motivation (Collins et al., 2010; Cooper & Holgersen, 2016), external motivators (e.g., Sturgess et al., 2016), supportive attitudes from the staff, and a safe environment (Stasch et al., 2018; Sturgess et al., 2016), as well as encouragement from family and staff (e.g., Drapeau et al., 2004; Kolton, 2002). The study, however, does not seem to reflect other findings, such as negative appraisal of the effectiveness of treatment and cynicism regarding economic motives of the correctional services (Brown & Tully, 2014; Mann et al., 2013).

### Methodological Reflections

The methodological design, where interviewees displaying various degrees of treatment motivation were included (in addition, readiness was examined both retrospectively and in the present), provides multi-facets of the studied phenomenon. Ideally, the non-participant group would be larger. However, it was challenging to recruit individuals from this group. Given that no other interview study, to my knowledge, has succeeded in this regard, it is probably a general research obstacle. The findings, especially the concept of looping disruption, may be transferable to other countries’ correctional services or other total institutions (mental hospitals, etcetera). Nevertheless, despite some anti-therapeutic elements demonstrated in this study, the Swedish Prison and Probation Service is strongly rehabilitation-oriented. These processes may not be similar in other countries or when studying other groups or contexts, e.g., women or probationers; hence, more studies in other contexts are needed.

This study does not objectively capture events or processes and single coder studies always include a risk of bias. Instead, it is based on meaning-making and interpretation, building on interviewees’ experiences and recapitulation of events. Furthermore, the critical realist position postulates that mechanisms in the “real domain” cannot be observed (Benton & Craib, 2011); hence, the proposed concept of looping disruption merely constitutes a hypothesis and/or possibly an analytical tool. Ethnographic studies might be preferable when studying institutional and therapeutic processes. However, this was not deemed to be feasible, due to the ethical and security constraints in the prison context. Furthermore, a service user perspective, using the lived experience of a highly stigmatized group, such as those who have sexually offended, can be ethically warranted. Future research should investigate how looping can be mitigated, besides the relational processes examined in this paper. In addition, studies of similar phenomena in other total institutions should be conducted in order to explore if the proposed concept of *looping disruption* could serve as a useful analytical tool to understand readiness in a broader, general sense.

## Notes

1. This group is often referred to as “refusers”. However, I chose the more neutral term non-participants, since motivation often fluctuates.

2. Words emphasized by the interviewee are noted by the use of capital letters.

3. Ardalan had started treatment but then discontinued; at the time of the interview, he had changed his mind again and resumed treatment.

4. It is noteworthy that several of the interviewees in the current study were not exclusively convicted of sexual offenses.
